# Comparison of Neuro‐cognitive profile of first degree relatives of Alzheimer’s Dementia with other major psychiatric illness

**DOI:** 10.1002/alz.089199

**Published:** 2025-01-03

**Authors:** Arushi Singh, Pavithra Dayal, Mahashweta Bhattacharya, Mino Susan Joseph, Alka Shaktan, Pramod Kumar, Sreenivasulu Mallappagari, Shruthi Narayana, Moorthy Muthukumaran, Alagarsami A R, Geetha C V, Pratibha Vinod, Vasundhra Teotia, Farooq Ali Syed, Shridhar Utagi, Pavithra Jayasankar, ADBS Consortium

**Affiliations:** ^1^ National Institute of Mental Health and Neurosciences (NIMHANS), Bengaluru, Karnataka India; ^2^ National Institute of Mental Health and Neurosciences (NIMHANS), Bengaluru, Karnataka India; ^3^ National Institute of Mental Health and Neurosciences. (NIMHANS), Bengaluru, Karnataka India; ^4^ National Institute of Mental Health and Neuroscienes (NIMHANS), Bengaluru, Karanataka India; ^5^ National Institute of Mental Health and Neuroscienes (NIMHANS), Bengaluru, Karnataka India; ^6^ National Institute of Mental Health and Neurosciences, Bangalore, Karnataka India

## Abstract

**Background:**

First‐degree relatives of patients suffering from Alzheimer’s Dementia (AD) are at increased risk for developing dementia, yet the associations between family history of AD and cognitive dysfunction remain unclear. Our study aims to understand the intricate interplay between familial risk factors and neurocognitive functioning in AD FDRs versus FDRs of other major psychiatric illnesses by comparing the neuro‐ cognitive functions of unaffected first‐degree relatives (FDR) of patients with AD with unaffected FDR of other major psychiatric illness including Schizophrenia(SCHIZ), Substance use disorders(SUD), Obsessive compulsive disorders(OCD) and Bipolar disorder(BPAD). Subsequently, we also compare the Neuro cognitive performance of FDR’s of AD at baseline and after two years longitudinally.

**Method:**

499 unaffected FDRs (AD‐ 100, SUD – 106, BPAD – 103, OCD ‐94, SCHIZ – 96) were recruited who underwent a comprehensive neuropsychogical battery. The battery includes Digit Span Forward (DSF), Auditory Verbal Learning Test (AVLT), N Back test (NBT) and Color trials (CT) to test for attention, verbal learning, working memory and executive function respectively. Assessments were done twice – at baseline and after two years for FDRs of AD (N = 32). Potential effect modifiers were controlled and generalised linear model was used to compare the neurocognitive functioning of different cohorts. Analysis was adjusted for years of education and gender. Wilcoxon Signed rank test was used to compare the baseline and follow up neurocognitive parameters of AD cohort.

**Result:**

FDRs of AD performed significantly worse in the domains of sustained attention, phonological memory and learning capacity as compared to the other cohorts. However, they performed significantly better in the tests covering verbal working memory, storage capacity and mental flexibility. No significant difference was found when neuro‐ cognitive parameters of FDRs of AD were compared at baseline and 2 years apart.

**Conclusion:**

The current findings provide initial evidence suggesting unsatisfactory performance of the AD FDRs in few domains as compared to others, however the stability of neurocognitive measures longitudinally over a two‐year period suggests the need for further exploration into the complex reciprocity of familial risk factors and cognitive outcomes in at‐risk populations.

